# Supplementary material for: Supramammillary glutamate neurons are a key node of the arousal system
Source: Nat Commun. 2017 Nov 10;8:1405. doi: 10.1038/s41467-017-01004-6 (PMC5680228; doi:10.1038/s41467-017-01004-6)
Supplement: Supplementary file 2 — Descriptions of Additional Supplementary Files [file 41467_2017_1004_MOESM2_ESM.pdf]

Description of Additional Supplementary Files

File Name: Supplementary Movie 1
Descriptions: Active wake was noted for the following hours with a normal repertoire of behavior, but without the napping that is typical during the light period.
